# *PIWIL1* gene polymorphism and pediatric acute lymphoblastic leukemia relapse susceptibility among Chinese children: a five-center case–control study

**DOI:** 10.3389/fonc.2023.1203002

**Published:** 2023-11-02

**Authors:** Wenjiao Ding, Dao Wang, Mansi Cai, Yaping Yan, Shanshan Liu, Xiaodan Liu, Ailing Luo, Decheng Deng, Xiaoping Liu, Hua Jiang

**Affiliations:** ^1^ Department of Hematology and Oncology, Guangzhou Women and Children’s Medical Center, Guangzhou Medical University, Guangdong Province Clinical Research Center for Child Health, Guangzhou, Guangdong, China; ^2^ Department of Pediatrics, The First Affiliated Hospital of Zhengzhou University, Zhengzhou, China; ^3^ Division of Birth Cohort Study, Guangzhou Women and Children’s Medical Center, Guangzhou Medical University, Guangdong Provincial Clinical Research Center for Child Health, Guangzhou, China

**Keywords:** acute lymphoblastic leukemia, PIWIL1, piRNA, polymorphism, relapse susceptibility

## Abstract

**Objective:**

*PIWIL1* polymorphisms’ role in pediatric acute lymphoblastic leukemia (ALL) relapse susceptibility remains undiscovered.

**Methods:**

A case–control designed and multiple logistic regression model was performed to evaluate the overall risk of pediatric ALL and five single-nucleotide polymorphisms (SNPs) of *PIWIL1* gene (rs35997018 C>T, rs1106042 A>G, rs7957349 C>G, rs10773771 C>T, and rs10848087 A>G) in 785 cases and 1,323 controls, which were genotyped by TaqMan assay. The odds ratio (OR) and its 95% confidence interval (CI) were used to estimate the relationship. Stratified analysis was used to investigate the correlation of rs1106042 and rs10773771 genotypes and pediatric ALL relapse susceptibility in terms of age, sex, number of white blood cells (WBC), immunophenotyping, gene fusion type, karyotype, primitive/naïve lymphocytes, and minimal residual disease (MRD) in bone marrow. Finally, the haplotype analysis was performed to appraise the relationship between inferred haplotypes of *PIWIL1* and pediatric ALL risk.

**Results:**

Among the five analyzed SNPs, rs1106042 A>G was related to increased ALL risk, and rs10773771 C>T was related to decreased ALL risk. Compared to the GG genotype, the rs1106042 GA/AA had a deleterious effect on children of age <120 months, who were female and male, had high or average number of WBC, pro-B ALL, pre-B ALL, T-ALL, low- and middle-risk ALL, E2A-PBX fusion gene, non-gene fusion, abnormal diploid, high hyperdiploid, hypodiploid, and normal diploid. Moreover, rs1106042 A>G harmfully affected primitive/naïve lymphocytes and MRD on days 15–19, day 33, and week 12. On the contrary, rs10773771 TC/CC exhibited a protective effect on ALL children with the TEL-AML fusion gene. Haplotype analysis demonstrated that haplotypes CAGT, TACC, TACT, and TAGT were significantly associated with increased pediatric ALL relapse susceptibility.

**Conclusion:**

*PIWIL1* rs1106042 A>G was related to increased ALL risk, and rs10773771 C>T was linked to decreased ALL risk in eastern Chinese children. Rs1106042 GA/AA may predict poor prognosis.

## Introduction

Acute lymphoblastic leukemia (ALL) is a malignant tumor with the highest morbidity, accounting for approximately 25% of cancer diagnoses in children under the age of 15 years (1). With therapeutics’ development, pediatric ALL’s long-term survival rate has reached more than 90%. However, nearly 10%–20% of ALL children relapse after initial complete remission. The mechanism of relapse susceptibility of pediatric ALL is still undiscovered. More and more data have shown that genetic variations, including single-nucleotide polymorphisms (SNPs), correlate closely with ALL relapse (2). However, they are insufficient to promote the initiation and relapse of ALL. Recently, epigenetic alterations have been considered to contribute to the development of ALL and ultimately disease relapse (3). We first analyzed the correlation between PIWIL1 SNPs and the risk of ALL in children and then analyzed the correlation between PIWIL1 SNPs and the recurrence of ALL in children by stratification.

Non-coding RNAs (ncRNAs) are divided into linear RNAs and circular RNAs. Based on the size and location, linear ncRNAs are classified into several types including transfer RNAs, ribosome RNAs, small nucleolar RNAs, small nuclear RNAs, microRNAs, long non-coding RNAs, and PIWI-interacting RNAs (piRNAs) (4). PiRNAs were discovered in 2006; they are single-stranded RNAs with a length of 26–31 nucleotides (5). Pre-piRNAs could be recognized and bound by P-element-induced wimpy testis proteins (PIWI) to form the piRNA-induced silencing complex (piRISC). PiRISC plays an important role in silencing transposable elements. Some piRNAs have been verified as tumor suppressors or oncogenes. PIWI proteins were described to participate in cell proliferation, apoptosis, and metastasis in multiple cancers (6, 7).

PiRNAs are 25–31 nucleotides in length and comprise non-coding RNAs interacting with Argonaute family proteins (i.e., PIWI family proteins) to regulate gene expression. So far, four PIWI family proteins have been identified in humans: PIWIL1 (HIWI), PIWIL2 (HILI), PIWIL3, and PIWIL4 (HIWI2). Among the PIWI family, the most studied cancers were colorectal cancer and breast cancer, and PIWIL1 is the most studied protein among the PIWI family (8).

Moreover, *PIWIL1* polymorphisms were reported to be associated with human diseases. In the PIWI family protein systematic review and meta-analysis, a significantly higher risk of mortality with higher piwil1/PIWIL1 expression was found in some reported cancers, some investigations showed no significant difference, and one showed a lower risk of death. In the reanalysis of the Human Protein Pathology Atlas (9), piwil1 showed the largest range of expression. A relatively large number of samples across cancer types exhibited an expression of >20 FPKM. Similar to piwil1’s expression pattern in normal tissues, testicular cancer had the largest proportion of samples with high piwil1 expression. Most cancer types displayed very low piwil1 expression (i.e., very few samples with an expression of >5 FPKM) (8). *PIWIL1* rs10773771 C>T is associated with a risk of large artery atherosclerosis stroke (10). Rs28416520 in *PIWIL1* gene promoter region is related to an enhanced risk of gastric cancer (11). *PIWIL1* rs1106042 showed positive correlations with non-obstructive azoospermia in the Han population of Northeast China (12).

PiRNA’s role in leukemia received a great deal of interest. A subpopulation of piRNAs may have a significant impact on epigenetic mechanisms, including heterochromatin formation, histone modifications, post-transcriptional modifications, and polycomb group-mediated transgene silencing (13, 14). In a CML cell line K562, overexpression of Hiwi produced tumors in BALB/c nude mice smaller than the control group (15). In the U937 cell line, overexpressing piRNA 011186 accelerated the cell cycle, reduced apoptosis, and inhibited CDKN2B gene expression (16). AML may have new prognostic and diagnostic biomarkers such as piRNA 32877 and piRNA 33195 (17). In multiple myeloma patients and cell lines, piRNA-823 was elevated and linked with the clinical stage. By controlling DNA methylation and angiogenesis in multiple myeloma, piRNA-823 aids in carcinogenesis (18). Until now, not only the expression and function of PIWIL1 in ALL but also the correlation between *PIWIL1* polymorphisms and ALL risk is still unclarified.

In the present study, a total of five SNPs were selected to evaluate the relationship between *PIWIL1* polymorphisms and pediatric ALL. The correlation between *PIWIL1* polymorphisms and clinical parameters of pediatric ALL was analyzed. The current study was a case–control study that was performed using samples from East China.

## Materials and methods

### Patients and healthy controls

A total of 2,118 children from the Children’s Hospital of Nanjing Medical University were enrolled in this study. The child participants comprised 785 ALL children and 1,323 healthy children, with an age range of 6 to 204 months. ALL cases were collected from Guangzhou Women and Children’s Medical Center, Guangzhou Medical University; The First Affiliated Hospital, Sun Yat-sen University; Sun Yat-sen Memorial Hospital, Sun Yat-sen University; Nanfang Hospital, Southern Medical University; and Zhujiang Hospital, Southern Medical University, during January 2017 to May 2019. Written consent was obtained from all participants at enrolment into the study, and ethics committee approval was obtained prior to the study from the institutional review board of Guangzhou Women and Children’s Medical Center, Guangzhou Medical University. All patients were diagnosed according to standard methods, including cytomorphologic, cytochemical, and immunophenotyping methods. All enrolled ALL children had not accepted previous therapy or transplantation and were treated with CCCG-ALL-2015 or CCLG ALL 2018 protocol in five centers. Some relapsed children underwent transplantation, but unfortunately, we did not have statistics. Control cases were randomly selected from the volunteers visiting the hospital and matched according to the expected age and gender distribution of the ALL group. The study was approved by the institutional ethics committee of Guangzhou Women and Children’s Medical Center, Guangzhou Medical University, and written informed consent was acquired from all participants in accordance with the Declaration of Helsinki.

### SNP selection

National Center for Biotechnology Information (NCBI) dbSNP database (http://www.ncbi.nlm.nih.gov/projects/SNP) and SNP info (http://snpinfo.niehs.nih.gov/snpfunc.htm) online software were used to select the SNPs in *PIWIL1* gene. The selected SNPs should fulfill the following criteria: 1) the minor allele frequency (MAF) was >5% of Chinese Han subjects in HapMap and 2) located in the exon, 5′ untranslated regions (5′ UTR), 3′ UTR, 5′ flanking region, and exon of *PIWIL1* gene, which were predicted to have potential functional; 3) each SNP should be in low linkage disequilibrium (R^2^ < 0.8). The probes were bought from Guangzhou Angke Biotechnology Co., Ltd., by TaqMan SNP assays mto human sm 10. The details are described in the “qRT-PCR and Genotyping” section. Finally, five SNPs in the *PIWIL1* gene (rs35997018 C>T, rs1106042 A>G, rs7957349 C>G, rs10773771 C>T, and rs10848087 A>G) were chosen.

### qRT-PCR and genotyping

With the use of TRIzol RNA extraction, extracted reagent RNA and the concentration and purity of the extracted RNA were determined by NanoDrop (Thermo Scientific NanoDrop 2000 spectrophotometer, Waltham, MA, USA). With the use of the Life Pro PCR instrument, cDNA and amplified samples were obtained using fluorescence quantitative PCR (ABI 7500 real-time fluorescent quantitative PCR system, USA) with qPCR system. After the completion of PCR, the threshold line was set according to the actual situation, and the amplification curve was analyzed. The BCR/ABL1 and ABL1 standard curves were used to determine the BCR/ABL1, TEL-AML copy number, and ABL1 copy number of the specific specimen, and the negative and positive ratios were calculated by Kangshengda Medical Laboratory Co., Ltd., Wuhan, China.

Genomic DNA was extracted from whole blood using the DNA extraction kit (Tiangen, Beijing, China) following the manufacturer’s instructions. TaqMan genotyping kits (Tiangen, Beijing, China) were used for SNP genotyping on an ABI 7900 (Applied Biosystem, Foster City, CA, USA). Genotyping results were confirmed by randomly assaying 10% of the original specimens for replication to exclude genotyping errors. There were no discrepancies between genotypes determined in duplicate.

### Statistical analyses

The compliance of genotypes with the Hardy–Weinberg equilibrium (HWE) among controls was tested using a goodness-of-fit *χ* ^2^ test. Data were statistically analyzed using the *χ* ^2^ test to ascertain differences in alleles, genotype, and haplotype frequencies. The age- and gender-adjusted odds ratio (ORs) and 95% confidence interval (CIs) for the relationships between the SNPs and ALL risk were determined by multivariate logistic regression analysis. All statistical analyses were conducted using SAS v10.0 (SAS Institute, Cary, NC, USA). In the present study, all *p*-values were two-sided, and values of *p* < 0.05 were considered statistically significant.

## Results

### Population characteristics

The clinical characteristic data of pediatric ALL samples and healthy controls are described in **Table 1**. There was no significant difference between the ALL group and the control group for Eastern Chinese children regarding age (*p* = 0.453) and gender (*p* = 0.278). In the ALL group, the age of children was from 6 to 204 months, 659 (83.95%) children were younger than 120 months, and 84 (10.70%) children were older than 120 months; 298 cases were girls and 435 cases were boys; the number of white blood cells in 334 cases (42.55%) was ≥10 × 10^9^/L, and in 377 (48.03%), it was normal; 223 cases (28.41%) were pro-B-cell ALL, 166 cases (21.15%) were pre-B-cell ALL, 293 cases (37.32%) were common B-cell ALL, 67 cases (8.54%) were T-cell ALL, and 5 cases (0.64%) were mixed-type ALL. As to gene fusion type, 27 cases (3.44%) were BCR-ABL type, 24 cases (3.06%) were E2A-PBX type, 16 cases (2.04%) were MLL type, 7 cases (0.89%) were SIL-TAL type, 9 cases (1.15%) were TCF3-PBX1 type, 7 cases were TEL/ETV6 type, 87 cases (11.08%) were TEL-AML type, 65 cases (8.28%) were other types, and 140 cases (17.83) had no gene fusion. There were 229 low-risk cases (29.17%), 31 normalized-risk cases (3.95%), 368 middle-risk cases (46.88%), and 87 high-risk cases (11.08%). Regarding karyotype, 231 cases (29.43%) were normal diploid, 66 cases (8.4%) were high hyperdiploid, 29 cases (3.69%) were low hyperdiploid, 25 cases (3.18%) were hypodiploid, and 178 cases (22.68%) were abnormal diploid. The ratio of primitive/naïve lymphocytes and minimal residual disease (MRD) in marrow on days 15–19, day 33, and week 12 after chemotherapy was calculated. On days 15–19, primitive/naïve lymphocytes of 38 cases (4.84%) were <0.5%, and those of 511 cases (65.10%) were ≥0.5%; the MRD of 45 cases (5.73%) was <0.01%, and that of 447 cases (56.94%) was ≥0.01%. On day 33, primitive/naïve lymphocytes of 65 cases (8.28%) were <0.5%, and those of 466 cases (59.36%) were ≥0.5%; the MRD of 275 cases (35.03%) was <0.01%, and that of 240 cases (30.57%) was ≥0.01%. On week 12, primitive/naïve lymphocytes of 26 cases (3.31%) were <0.5%, and those of 313 cases (39.87%) were ≥0.5%; the MRD of 308 cases (39.24%) was <0.01%, and that of 30 cases (3.82%) was ≥0.01%. In the ALL group, 29 cases (3.69%) relapsed, and 539 cases (68.66%) did not.

**Table 1:** Frequency distribution of selected characteristics in pediatric ALL cases and cancer-free controls.

Variables	Cases (n = 785)		Controls (n = 1,323)		*p* ^a^
	No.	%	No.	%	
Age range, months	6–204		11–180		
Mean ± SD					0.453
<120	659	83.95	1,139	86.09	
≥120	84	10.70	129	9.75	
NA	42	5.35	55	4.16	
Gender					0.278
Female	298	37.96	503	38.02	
Male	435	55.41	765	57.82	
NA	52	6.62	55	4.16	
WBC					
>10	334	42.55			
≤10	377	48.03			
NA	73	9.30			
Immunophenotyping					
Pro-B	223	28.41			
Pre-B	166	21.15			
Common B	293	37.32			
T-ALL	67	8.54			
Mix	5	0.64			
NA	31	3.95			
Gene fusion type					
BCR-ABL	27	3.44			
E2A-PBX	24	3.06			
MLL	16	2.04			
SIL-TAL	7	0.89			
TCF3-PBX1	9	1.15			
TEL/ETV6	7	0.89			
TEL-AML	87	11.08			
Others	65	8.28			
Non	140	17.83			
NA	403	51.34			
Risk level					
Low	229	29.17			
Normalized	31	3.95			
Middle	368	46.88			
High	87	11.08			
NA	70	8.92			
Karyotype					
Abnormal diploid	231	29.43			
High hyperdiploid	66	8.41			
Low hyperdiploid	29	3.69			
Hypodiploid	25	3.18			
Normal diploid	178	22.68			
NA	256	32.61			
Primitive/naïve lymphocytes in marrow (%, 15–19 days)					
<0.5	38	4.84			
≥0.5	511	65.10			
NA	236	30.06			
MRD in marrow (%, 15–19 days)					
<0.01	45	5.73			
≥0.01	447	56.94			
NA	293	37.32			
Primitive/naïve lymphocytes in marrow (%, 33 days)					
<0.5	65	8.28			
≥0.5	466	59.36			
NA	254	32.36			
MRD in marrow (%, 33 days)					
<0.01	275	35.03			
≥0.01	240	30.57			
NA	270	34.39			
Primitive/naïve lymphocytes in marrow (%, 12 weeks)					
<0.5	26	3.31			
≥0.5	313	39.87			
NA	446	56.82			
MRD in marrow (%, 12 weeks)					
<0.01	308	39.24			
≥0.01	30	3.82			
NA	448	57.07			
Relapse					
−	539	68.66			
+	29	3.69			
NA	217	27.64			

SD, standard deviation; NA, not available; WBC, white blood cells; ALL, acute lymphoblastic leukemia; MRD, minimal residual disease.

a Two-sided χ^2^ test for distributions between ALL cases and cancer-free controls.

### Correlation of *PIWIL1* gene polymorphisms with pediatric ALL risk

The relationship between genotype frequencies of the *PIWIL1* gene and pediatric ALL risk was evaluated (**Table 2**). First, for the single-locus analysis, the HWE of rs35997018 C>T, rs1106042 A>G, rs7957349 C>G, rs10773771 C>T, and rs10848087 A>G in control populations was calculated. However, rs10848087 did not conform to the HWE (<0.001). Then, the correlation of the other four SNPs in *PIWIL1* gene with ALL risk was analyzed. The carriers of the rs1106042 (AA *vs.* GG: adjusted OR = 5.623, 95% CI = 3.291–9.696, *p* < 0.001) and rs10773771 (TC *vs.* TT: adjusted OR = 0.744, 95% CI = 0.615–0.900, *p* = 0.002; CC *vs.* TT: adjusted OR = 0.741, 95% CI = 0.553–0.992, *p* = 0.044) variant alleles demonstrated significant increased risk of pediatric ALL. However, there was no relationship between rs35997018, 7957349, and pediatric ALL risk. We then defined rs1106042 GA/AA as the risk genotype and rs10773771 TC/CC as the protective genotype.

**Table 2:** Relationship between genotype frequencies of *PIWIL1* gene and pediatric ALL risk evaluation.

Genotype	Cases	Controls	*p* ^a^	Crude OR	*p*	Adjusted OR	*p* ^b^
	(N = 785)	(N = 1,323)		(95% CI)		(95% CI) bõ	
rs10848087 (HWE < 0.001)							
GG	561 (76.12)	912 (70.37)		1.000		1	
GA	116 (15.74)	306 (23.61)		0.585 (0.461–0.741)	<0.001	0.586 (0.462–0.742)	<0.001
AA	60 (8.14)	78 (6.02)		1.186 (0.834–1.686)	0.342	1.188 (0.836–1.690)	0.337
Additive			<0.001	0.903 (0.750–1.052)	0.191	0.905 (0.776–1.542)	0.199
Dominant	176 (23.88)	384 (29.63)	0.005	0.745 (0.606–0.917)	0.005	0.746 (0.607–0.918)	0.006
Recessive	677 (91.86)	1,218 (93.98)	0.067	1.384 (0.976–1.963)	0.068	1.392 (0.981–1.974)	0.064
rs1106042 (HWE = 0.267)							
GG	500 (70.22)	1,044 (80.43)		1.000		1	
GA	158 (22.19)	236 (18.18)		1.249 (0.997–1.565)	0.053	1.246 (0.994–1.562)	0.056
AA	54 (7.58)	18 (1.39)		5.597 (3.252–9.633)	<0.001	5.623 (3.291–9.696)	<0.001
Additive			<0.001	1.803 (1.561–2.144)	<0.001	1.802 (1.515–2.143)	<0.001
Dominant	212 (29.78)	254 (19.57)	<0.001	1.743 (1.411–2.153)	<0.001	1.741 (1.409–2.150)	<0.001
Recessive	658 (92.42)	1,280 (98.61)	<0.001	5.834 (3.394–10.028)	<0.001	5.865 (3.411–10.084)	<0.001
rs35997018 (HWE = 0.139)							
TT	399 (52.99)	649 (50.43)		1.000		1	
TC	296 (39.31)	545 (42.35)		0.963 (0.717–1.040)	0.121	0.865 (0.710–1.042)	0.126
CC	58 (7.70)	93 (7.23)		0.991 (0.699–1.406)	0.960	0.993 (0.700–1.408)	0.968
Additive			0.404	0.948 (0.821–1.095)	0.470	0.950 (0.822–1.096)	0.481
Dominant	354 (47.01)	638 (49.57)	0.264	0.903 (0.754–1.081)	0.264	0.904 (0.755–1.083)	0.273
Recessive	695 (92.30)	1,194 (92.77)	0.692	1.072 (0.762–1.508)	0.690	1.072 (0.762–1.508)	0.689
rs7957349 (HWE = 0.055)							
GG	432 (57.91)	765 (58.67)		1.000		1	
GC	268 (35.92)	451 (34.59)		0.989 (0.818–1.195)	0.710	0.986 (0.816–1.192)	0.887
CC	46 (6.17)	88 (6.75)		0.870 (0.598–1.265)	0.466	0.871 (0.599–1.266)	0.469
Additive			0.765	1.005 (0.868–1.162)	0.951	1.004 (0.867–1.162)	0.960
Dominant	314 (42.09)	539 (41.33)	0.738	1.032 (0.860–1.238)	0.738	1.030 (0.858–1.236)	0.751
Recessive	700 (93.83)	1,216 (93.25)	0.608	0.908 (0.828–1.313)	0.608	0.909 (0.629–1.314)	0.612
rs10773771 (HWE = 0.515)							
TT	275 (41.23)	496 (39.40)		1.000		1	
TC	307 (46.03)	597 (47.42)		0.744 (0.615–0.899)	0.002	0.744 (0.615–0.900)	0.002
CC	85 (12.74)	166 (13.19)		0.740 (0.553–0.992)	0.044	0.741 (0.553–0.992)	0.044
Additive			0.737	0.951 (0.828–1.093)	0.483	0.951 (0.828–1.093)	0.481
Dominant	392 (58.77)	763 (60.60)	0.435	0.927 (0.765–1.122)	0.435	0.926 (0.765–1.122)	0.433
Recessive	582 (87.26)	1,093 (86.81)	0.784	0.962 (0.727–1.273)	0.785	0.961 (0.727–1.272)	0.783

HWE, Hardy–Weinberg equilibrium.

a χ^2^ test for genotype distributions between leukemia cases and cancer-free controls.

b Adjusted for age and gender.

### Stratification analysis of rs1106042 and rs10773771 with ALL relapse susceptibility

The effect of rs1106042 A>G and rs10773771 C>T on age, gender, white blood cell (WBC) number, immunophenotyping, risk grade, gene infusion type, karyotype, primitive/naïve lymphocytes, MRD in marrow, and relapse was evaluated by subgroup analysis. As shown in **Table 3**, rs1106042 GA/AA had a harmful effect in children <120 months of age (adjusted OR = 1.770, 95% CI = 1.415–2.213, *p* < 0.001), girls (adjusted OR = 1.811, 95% CI = 1.280–2.563, *p* = 0.001), and boys (adjusted OR = 1.767, 95% CI = 1.333–2.342, *p* < 0.001). The rs1106042 GA/AA alleles were also identified to enhance the risk in children with WBC > 10 × 10^9^/L (adjusted OR = 1.610, 95% CI = 1.209–2.144, *p* = 0.001), WBC ≤ 10 × 10^9^/L (adjusted OR = 1.750, 95% CI = 1.337–2.292, *p* < 0.001), pro-B ALL (adjusted OR = 2.192, 95% CI = 1.580–3.041, *p* < 0.001), pre-B ALL (adjusted OR = 1.655, 95% CI = 1.132–2.419, *p* = 0.009), T-ALL (adjusted OR = 2.480, 95% CI = 1.437–4.280, *p* = 0.001), low-risk level (adjusted OR = 1.435, 95% CI = 1.015–2.028, *p* = 0.041), middle-risk level (adjusted OR = 2.031, 95% CI = 1.555–2.654, *p* < 0.001), E2A-PBX gene fusion (adjusted OR = 2.798, 95% CI = 1.130–6.926, *p* = 0.026), other gene fusion (adjusted OR = 3.611, 95% CI = 1.378–9.463, *p* = 0.009), no gene fusion (adjusted OR = 1.829, 95% CI = 1.229–2.721, *p* = 0.003), abnormal diploid (adjusted OR = 1.510, 95% CI = 1.080–2.113, *p* = 0.016), high hyperdiploid (adjusted OR = 1.916, 95% CI = 1.107–1.316, *p* = 0.020), hypodiploid (adjusted OR = 3.595, 95% CI = 1.438–8.986, *p* = 0.006), normal diploid (adjusted OR = 1.815, 95% CI = 1.257–2.619, *p* = 0.002), primitive/naïve lymphocytes in marrow ≥5% on days 15–19 (adjusted OR = 1.834, 95% CI = 1.441–2.334, *p* < 0.001), day 33 (adjusted OR = 1.834, 95% CI = 1.430–2.352, *p* < 0.001), and week 12 (adjusted OR = 1.949, 95% CI = 1.462–2.598, *p* < 0.001), primitive/naïve lymphocytes in marrow <5% on days 15–19 (adjusted OR = 1.568, 95% CI = 1.152–2.135, *p* = 0.004), day 33 (adjusted OR = 1.622, 95% CI = 1.214–2.165, *p* = 0.001), and week 12 (adjusted OR = 1.621, 95% CI = 1.262–2.082, *p* < 0.001), with MRD ≥ 0.01% on days 15–19 (adjusted OR = 1.579, 95% CI = 1.197–2.084, *p* = 0.001), on day 33 (adjusted OR = 2.264, 95% CI = 1.448–3.539, *p* < 0.001), with MRD < 0.01% on days 15–19 (adjusted OR = 1.887, 95% CI = 1.461–2.438, *p* < 0.001), on day 33 (adjusted OR = 1.670, 95% CI = 1.338–2.064, *p* < 0.001), and on week 12 (adjusted OR = 1.724, 95% CI = 1.394–2.133, *p* < 0.001), without relapse (adjusted OR = 1.774, 95% CI = 1.399–2.250, *p* < 0.001).

**Table 3:** Stratification analysis of rs1106042 and rs10773771 with ALL relapse susceptibility.

Variables	rs1106042		Adjusted OR^a^	*p* ^a^	rs10773771		Adjusted OR^a^	*p* ^a^
	(cases/controls)				(cases/controls)			
	GG	GA/AA	(95% CI)		TT	TC/CC	(95% CI)	
Age, months								
<120	447/1,044	189/254	1.770 (1.415–2.213)	<0.001	241/499	357/764	0.632 (0.350–1.143)	0.129
≥120	98/1,044	28/254	1.519 (0.797–2.895)	0.204	34/499	39/764	0.985 (0.805–1.205)	0.881
Gender								
Female	185/1,044	84/254	1.811 (1.280–2.563)	0.001	99/499	154/764	1.007 (0.732–1.385)	0.966
Male	279/1,044	113/254	1.767 (1.333–2.342)	<0.001	159/499	212/764	0.874 (0.680–1.123)	0.292
WBC								
>10	215/1,044	85/254	1.610 (1.209–2.144)	0.001	131/499	162/764	0.804 (0.622–1.039)	0.095
≤10	240/1,044	102/254	1.750 (1.337–2.292)	<0.001	120/499	192/764	1.042 (0.808–1.345)	0.749
Immunophenotyping								
Pro-B	128/1,044	67/254	2.192 (1.580–3.041)	<0.001	76/499	118/764	1.022 (0.749–1.394)	0.892
Pre-B	107/1,044	43/254	1.655 (1.132–2.419)	0.009	57/499	85/764	0.970 (0.680–1.382)	0.865
Common B	208/1,044	66/254	1.303 (0.856–1.775)	0.094	107/499	141/764	0.861 (0.653–1.134)	0.286
T-ALL	37/1,044	23/254	2.480 (1.437–4.280)	0.001	21/499	34/764	1.043 (0.595–1.828)	0.883
Mix	3/1,044	1/254	1.262 (0.129–12.328)	0.841	2/499	3/764	0.929 (0.154–5.616)	0.936
Risk								
Low	152/1,044	52/254	1.435 (1.015–2.028)	0.041	85/499	105/764	0.819 (0.601–1.116)	0.206
Normalized	18/1,044	8/254	1.868 (0.802–4.351)	0.148	8/499	12/764	1.003 (0.406–2.476)	0.995
Middle	222/1,044	310/254	2.031 (1.555–2.654)	<0.001	127/499	192/764	0.984 (0.765–1.266)	0.902
High	62/1,044	22/254	1.424 (0.857–2.365)	0.172	312/499	47/764	0.984 (0.615–1.572)	0.945
Gene fusion type								
BCR-ABL	19/1,044	7/254	1.436 (0.594–3.472)	0.421	8/499	15/764	1.218 (0.510–2.905)	0.657
E2A-PBX	12/1,044	8/254	2.798 (1.130–6.926)	0.026	8/499	11/764	0.905 (0.361–2.266)	0.831
MLL	11/1,044	5/254	1.791 (0.614–5.222)	0.286	7/499	9/764	0.827 (0.305–2.241)	0.709
SIL-TAL	5/1,044	2/254	1.552 (0.297–8.102)	0.602	4/499	2/764	0.327 (0.060–1.796)	0.199
TCF3-PBX1	6/1,044	1/254	0.635 (0.075–5.350)	0.676	4/499	5/764	0.776 (0.206–2.926)	0.708
TEL/ETV6	6/1,044	0/254	<0.001 (<0.001, >999.999)	0.960	3/499	2/764	0.442 (<0.074–2.656)	0.372
TEL-AML	60/1,044	21/254	1.446 (0.863–2.422)	0.162	39/499	36/764	0.609 (0.382–0.972)	0.037
Others	9/1,044	8/254	3.611 (1.378–9.463)	0.009	5/499	11/764	1.409 (0.486–4.085)	0.528
Non	90/1,044	40/254	1.829 (1.229–2.721)	0.003	51/499	62/764	0.795 (0.540–1.172)	0.247
Karyotype								
Abnormal diploid	153/1,044	56/254	1.510 (1.080–2.113)	0.016	78/499	121/764	1.089 (0.743–1.370)	0.955
High hyperdiploid	43/1,044	20/254	1.916 (1.107–1.316)	0.020	21/499	33/764	1.024 (0.586–1.791)	0.933
Low hyperdiploid	21/1,044	4/254	0.772 (0.262–2.273)	0.639	11/499	14/764	0.816 (0.367–1.815)	0.618
Hypodiploid	10/1,044	9/254	3.595 (1.438–8.986)	0.006	11/499	9/764	0.528 (0.217–1.284)	0.159
Normal diploid	109/1,044	48/254	1.815 (1.257–2.619)	0.002	65/499	87/764	0.877 (0.624–1.233)	0.451
Primitive/naïve lymphocytes in marrow (%, 15–19 days)								
<5	182/1,044	70/254	1.568 (1.152–2.135)	0.004	100/499	138/764	0.899 (0.678–1.191)	0.456
≥5	318/1,044	142/254	1.834 (1.441–2.334)	<0.001	175/499	254/764	0.958 (0.767–1.197)	0.707
MRD in the marrow (%, 15–19 days)								
<0.01	261/1,044	120/254	1.887 (1.461–2.438)	<0.001	146/499	215/764	0.961 (0.757–1.220)	0.746
≥0.01	239/1,044	92/254	1.579 (1.197–2.084)	0.001	129/499	181/764	0.912 (0.708–1.175)	0.476
Primitive/naïve lymphocytes in marrow (%, 33 days)								
<5	210/1,044	83/254	1.622 (1.214–2.165)	0.001	117/499	164/764	0.917 (0.705–1.193)	0.520
≥5	290/1,044	129/254	1.834 (1.430–2.352)	<0.001	158/499	232/764	0.958 (0.759–1.207)	0.714
MRD in the marrow (%, 33 days)								
<0.01	440/1,044	179/254	1.670 (1.338–2.064)	<0.001	246/499	345/764	0.916 (0.751–1.117)	0.385
≥0.01	60/1,044	33/254	2.264 (1.448–3.539)	<0.001	29/499	51/764	1.142 (0.714–1.827)	0.580
Primitive/naïve lymphocytes in marrow (%, 12 weeks)								
<5	311/1,044	123/254	1.621 (1.262–2.082)	<0.001	169/499	239/764	0.922 (0.735–1.158)	0.485
≥5	189/1,044	89/254	1.949 (1.462–2.598)	<0.001	106/499	157/764	0.966 (0.736–1.267)	0.803
MRD in the marrow (%, 12 weeks)								
<0.01	493/1,044	207/254	1.724 (1.394–2.133)	<0.001	274/499	386/764	0.920 (0.760–1.114)	0.393
≥0.01	7/1,044	5/254	2.864 (0.899–9.127)	0.075	1/499	10/764	6.258 (0.797–49.138)	0.081
Relapse								
−	340/1,044	147/254	1.774 (1.399–2.250)	<0.001	182/499	276/764	0.985 (0.791–1.226)	0.891
+	17/1,044	8/254	1.870 (0.794–4.404)	0.152	14/499	12/764	0.559 (0.256–1.222)	0.145

ALL, acute lymphoblastic leukemia; WBC, white blood cells; MRD, minimal residual disease.

a Adjusted for age and gender.

On the contrary, rs10773771 TC/CC alleles exhibited a protective effect on children with TEL-AML gene fusion (adjusted OR = 0.609, 95% CI = 0.382–0.972, *p* = 0.037).

### Haplotype analysis of SNPs in *PIWIL1* correlated with pediatric ALL expression

Furthermore, whether the haplotypes of *PIWIL1* rs1106042 A>G, rs7957349 C>G, rs10773771 C>T, and rs35997018 C>T are linked to pediatric ALL risk were calculated. The wild-type allele TGGT was defined as the control allele. The results showed that haplotypes CAGT (adjusted OR = 3.781, 95% CI = 1.556–2.726, *p* = 0.003), TACC (adjusted OR = 1.700, 95% CI = 1.239–2.333, *p* = 0.001), TACT (adjusted OR = 5.945, 95% CI = 2.729–12.953, *p* < 0.001), and TAGT (adjusted OR = 9.306, 95% CI = 4.445–19.485, *p* < 0.001) would increase ALL relapse susceptibility in Chinese children (**Table 4**).

**Table 4:** Haplotype analysis of SNPs in PIWIL1 correlated with pediatric ALL expression.

Haplotypes	Cases (n = 1,166)	Controls (n = 2,436)	Crude OR (95% CI)	*p*	Adjusted OR (95% CI)	*p*
	No.%	No.%				
TGGT	438 (37.56)	1,019 (41.83)	1.000		1.000	
CACC	6 (0.51)	29 (1.19)	0.481 (0.198–1.168)	0.106	0.480 (0.198–1.164)	0.105
CACT	14 (1.20)	0	>999.999 (<0.001, >999.999)	0.950	>999.999 (<0.001, >999.999)	0.950
CAGC	12 (1.03)	21 (0.86)	1.329 (0.648–2.726)	0.437	1.326 (0.646–2.720)	0.441
CAGT	13 (1.11)	8 (0.33)	3.781 (1.556–9.186)	0.003	3.860 (1.588–9.384)	0.003
CGCC	27 (2.32)	79 (3.24)	0.795 (0.506–1.248)	0.319	0.796 (0.507–1.250)	0.322
CGCT	19 (1.63)	74 (3.04)	0.597 (0.356–1.001)	0.051	0.600 (0.358–1.005)	0.052
CGGC	105 (9.01)	239 (9.81)	1.022 (0.792–1.319)	0.867	1.028 (0.796–1.328)	0.830
CGGT	130 (11.15)	244 (10.02)	1.240 (0.975–1.577)	0.080	1.243 (0.977–1.581)	0.077
TACC	76 (6.52)	104 (4.27)	1.700 (1.239–2.333)	0.001	1.704 (1.242–2.339)	0.001
TACT	23 (1.97)	9 (0.37)	5.945 (2.729–12.953)	<0.001	6.000 (2.753–13.076)	<0.001
TAGC	43 (3.69)	76 (3.12)	1.316 (0.891–1.945)	0.168	1.309 (0.885–1.934)	0.177
TAGT	36 (3.09)	9 (0.37)	9.306 (4.445–19.485)	<0.001	9.238 (4.411–19.350)	<0.001
TGCC	43 (3.69)	114 (4.68)	0.878 (0.607–1.268)	0.487	0.870 (0.602–1.258)	0.460
TGCT	72 (6.17)	172 (7.06)	0.974 (0.724–1.311)	0.861	0.975 (0.724–1.312)	0.866
TGGC	109 (9.35)	239 (9.81)	1.061 (0.824–1.366)	0.646	1.063 (0.825–1.369)	0.626

SNPs, single-nucleotide polymorphisms; ALL, acute lymphoblastic leukemia.

## Discussion

In the present case–control study, we explored the possible relationship between *PIWIL1* gene polymorphisms and pediatric ALL sensitivity in eastern China. We identified that two of the five selected SNPs were associated with pediatric ALL risk: rs1106042 A>G was related to increased ALL risk, and rs10773771 C>T was related to decreased ALL risk. As far as we know, this is the first study on the association between the genetic variations of *PIWIL1* and pediatric ALL risk.

Increasing evidence has shown that PIWIL1 is upregulated in several tumors (19), and it is positively correlated with histological grade, clinical stage, and poor prognosis (20). PIWIL1 inhibits the invasion and migration abilities of pancreatic stellate cells through the PI3K/AKT/mTOR signaling pathway (21). It also appears to be involved in the carcinogenesis of pancreatic cancer (22). Knockdown of PIWIL1 could inhibit cell proliferation and induce apoptosis in glioma cells (23). PIWIL1 acts as a potential biomarker for predicting chemoresistance in cervical cancer (24). In pancreatic cancer, PIWIL1 promotes metastasis through interrupting cell–cell adhesion (25). Afterward, PIWIL1 could maintain self-renewal and survival of glioma stem cells (26). PIWIL1 is expressed in CD34+ stem/progenitor cells and is necessary for normal hematopoiesis in mice (27). As to leukemia, the function of PIWIL1 is only reported in chronic myeloid leukemia. Overexpression of PIWIL1 suppresses the proliferation of K562 cells and induces chemosensitivity to daunomycin (28). Wang et al. reported that *PIWIL1* rs10773771 C>T may be associated with a decreased risk of large artery atherosclerosis stroke, and then they inferred that rs10773771 could modify the mRNA secondary structure of *PIWIL1* and regulate miRNAs binding to the 3′-UTR of PIWIL1 by using bioinformatic analysis (10). The frequency of the GG genotype at rs28416520 locus shows a remarkably higher association with gastric cancer (11).

In the present study, we first genotyped five SNPs of *PIWIL1*, rs35997018 C>T, rs1106042 A>G, rs7957349 C>G, rs10773771 C>T, and rs10848087 A>G, and discovered that rs1106042 A>G was related to increased ALL risk, rs10773771 C>T was related to decreased ALL risk, and the other three SNPs were not associated with ALL risk in eastern Chinese children. However, the HWE of rs10848087 A>G was less than 0.05, and the other two SNPs were not found to be associated with ALL risk. Furthermore, we estimated the *PIWIL1* polymorphisms preferentially predisposed to any pediatric ALL subtype. Compared to the GG genotype, the rs1106042 GA/AA had a deleterious effect on children of age <120 months, female and male. Next, we proved that rs1106042 GA/AA increased the pediatric ALL risk in children with high or normal number of WBC, pro-B ALL, pre-B ALL, T-ALL, low- and middle-risk ALL, E2A-PBX fusion gene, non-gene fusion, abnormal diploid, high hyperdiploid, hypodiploid, and normal diploid. E2A-PBX fusion gene has been reported to be positively correlated with multidrug resistance in ALL (29). Our data showed that rs1106042 GA/AA enhanced the risk of ALL children with E2A-PBX fusion gene, which might be a prognostic factor for pediatric ALL. Meanwhile, rs10773771 TC/CC decreased the risk of ALL children with TEL-AML fusion gene. TEL-AML is the most common genetic alteration in pre-B ALL children (30). Luo et al. identified that *METTL14* rs298982 GA/AA and rs1064034 TA/AA had a protective effect in children with the TEL-AML fusion gene (31). In this study, rs10773771 TC/CC also played as a protective allele in ALL patients with the TEL-AML fusion gene.

In stratification analysis, we intended to explore the correlation between clinical characteristics, response to chemotherapeutics, and *PIWIL1* polymorphisms. In the present study, rs1106042 GA/AA enhanced ALL risk in children with primitive/naïve lymphocytes in marrow <5% or ≥5%, and MRD in marrow <0.01% and ≥0.01% on days 15–19 of induction chemotherapy. Moreover, rs1106042 GA/AA enhanced ALL risk in children with primitive/naïve lymphocytes in marrow <5% or ≥5% and MRD in marrow <0.01% or ≥0.01% on day 33 of chemotherapeutic inducing. Furthermore, rs1106042 GA/AA improved ALL risk in children with primitive/naïve lymphocytes in marrow <5% or ≥5%, and MRD in marrow <0.01% on week 12 of chemotherapeutic inducing, as well as children without relapse. These results indicated that rs1106042 GA/AA was closely associated with therapeutic response.

According to previous publications, haplotypes of multiple SNPs instead of single-locus analysis heighten the power for mapping and characterizing disease-related genes (32). Here, we detected whether haplotypes of *PIWIL1* are correlated with pediatric ALL risk. The results revealed that haplotypes CAGT, TACC, TACT, and TAGT were significantly associated with increased pediatric ALL relapse susceptibility. It suggested that a stronger effect of haplotypes on the pediatric ALL risk existed.

However, there are still some limitations. First, only children in eastern China were enrolled, so a larger sample size and multiple centers should be included in the future. Second, this was a retrospective work, so information bias and selection bias may inevitably exist.

In conclusion, *PIWIL1* rs1106042 A>G was related to increased ALL risk, and rs10773771 C>T was related to decreased ALL risk in eastern Chinese children; rs1106042 GA/AA may predict poor prognosis.

## Data availability statement

The original data presented in the study are included in the article/supplementary material, further original data can be obtained by contacting the corresponding author/s.

## Ethics statement

The studies involving humans were approved by the Ethics Committee of Guangzhou Women and Children Medical Center No (2023) 117A01. The studies were conducted in accordance with the local legislation and institutional requirements. Written informed consent for participation in this study was provided by the participants’ legal guardians/next of kin. Written informed consent was obtained from the minor(s)’ legal guardian/next of kin for the publication of any potentially identifiable images or data included in this article.

## Author contributions

HJ conceived and designed the analysis; XPL performed the statistical analysis; WD wrote the manuscript; Other authors collected the data and performed the experimental analysis. All authors contributed to the article and approved the submitted version.
